# Tardive dyskinesia: apropos of a case. This is a case related to drug side effects, whose uniqueness lies in the time of onset of symptoms, Tardive dyskinesia is a drug-induced hyperkinetic movement disorder.

**DOI:** 10.1192/j.eurpsy.2023.2037

**Published:** 2023-07-19

**Authors:** R. F. Díaz

**Affiliations:** Psychiatry, Complejo Asistencial de Segovia, Segovia, Spain

## Abstract

**Introduction:**

Tardive dyskinesia is finally diagnosed, it is a drug-induced hyperkinetic movement disorder associated with the use of dopamine receptor blocking agents, including first and second generation antipsychotic drugs, metoclopramide and prochlorperazine. Typically, the first-generation antipsychotics with increased dopamine D2 receptor affinity are affiliated with a higher risk of inducing tardive dyskinesia.

The most common manifestations of TD involve spontaneous movements of the mouth and tongue, but the arms, legs, trunk, and respiratory muscles may also be affected. Less commonly, the prominent feature is dystonia involving a focal area of the body such as the neck. TD can be irreversible and lifelong, with significant negative impacts on psychological health and quality of life.

**Objectives:**

Clinical review and treatment approach for tardive dyskinesia.

**Methods:**

Clinical case and literature review.

**Results:**

A 54-year-old male comes due to involuntary movements of a month of evolution in the tongue and lips that “he cannot control” and generates significant discomfort and anxiety. He reports occipital headache and at the level of both temporomandibular joints that does not wake him up at night or change its characteristics with postural changes. Reviewing the treatment describe that the patient was in treatment for at least 6 months without being able to specify the end of treatment (January to June 2021) Clebopride-Climethicone. This finding inclines the diagnosis towards an orolingual dyskinesia probably secondary to Orthopramides. Discharge was decided with treatment and follow-up in Neurology and Psychiatry consultations.

**Conclusions:**

The diagnosis and management of tardive dyskinesia are best made with an interprofessional team. In most cases, the primary clinician may suspect the diagnosis during follow-up. Movement disorders like tardive dyskinesias are frequently aggravated by the use of drugs that block dopamine. In susceptible patients, even a single dose of an anti-dopaminergic drug can quickly develop disabling movement disorders.

Currently the american academy of neurology recommends few treatments such as tetrabenazine or clonazepam. The first treatment for tardive dyskinesia has recently been approved, such as Velbazine, a vesicular monoamine transport type 2 (VMAT2) inhibitor, the extent of its use remains to be seen.

**Disclosure of Interest:**

None Declared

